# Life in the New Hebrides
*"Bible Illustrations from the New Hebrides." By the Rev. John Inglis, D.D. (London: Thomas Nelson and Sons.)


**Published:** 1890-09-06

**Authors:** 


### Life in the New Hebrides.*

In certain circles it is quite the fashion now-a-days to speak hard and scornful words of the *nup-a-missionary*—to borrow the name which the New Hebrideans use to describe that body of devoted men who leave the land of their birth to dwell among strangers, and bring to them the blessings of civilization and of Christianity. And we are ready to admit that some of these hard words have been deserved—that the zeal of would-be converters of the heathen has not always been according to knowledge, and that to pit against the keen and cultured minds of the East a half-educated, narrow-minded, bigoted young dunderhead as the exponent of the religion of Christ, is to court defeat and ridicule. This we believe, however, to be a thing of the past; and meanwhile, whatever may be thought of missions to Buddhists and Mahommedans, no one surely can deny that those who are labouring to civilise and Christianise the inhabitants of the South Sea Islands are doing a great work, both for those islanders and for the world in general.

In advocating the claims of his mission, Dr. Inglis cannily reminds us that "it is only at Christianised islands that any ship can calculate on obtaining supplies"; and now that the Pacific is becoming one of the great thoroughfares of the world, this is an important consideration. And if it is a great thing for our sailors to obtain supplies, it is a still greater thing to be sure that they themselves will not supply the larders of the inhabitants of islands to which they may become involuntary visitors. It was only in May last that a telegram appeared in one of the daily papers, announcing that fifty-one of the crew and passengers of a schooner which had been driven upon the reefs of Mallicolo (one of the New Hebrides group of islands) had been killed, roasted, and eaten, amid horrible scenes of cannibalistic rejoicing. We can only hope that as this tale had passed through the magnifying medium of the American press, it may be taken with a good many grains of salt; but the fact remains, that the New Hebrideans are in their natural state cannibals; and to transform the cannibal into a civilized, God-fearing, peaceable man, as we cannot doubt has been done on Aneityum, the principal scene of the missionaries' labours, is to render real service to the world.

It is only the first few chapters of Dr. Inglis' book which are taken up with his "Bible Illustrations"; the rest of the volume contains sketches of natural history, and of the manners and customs of the natives, to us the most interesting part of the work. Still, his parallels between Bible and New Hebridean incidents or sayings are sometimes instructive as well as curious. The story of Benjamin's mess becomes quite natural and explicable when we read of a feast on Aneityum given by the chief of the district to the missionary party. On the opening of the oven—a pit in which layers of food, of leaves, and of red-hot stones are placed alternately—the whole quantity of food was divided into four portions, the largest being for the Scotch missionaries, the two next in size for less important guests, and the last—which was about as much as those to whom it was allotted could eat in a sitting—for the givers of the feast. The missionaries' portion consisted of "two large baskets of taro, two fowls, a large fish in shape and size like a goodly 14-lb. salmon, a large bunch of bananas, fully ripe and yellow, and a large basket of fresh cocoa-nuts, husked, and ready for drinking." After the guests had finished their meal, their native servants gathered up all that was left, and packed it in baskets, carrying home enough to provide a sumptuous breakfast for themselves, their masters, and the natives living near at hand. "To have left anything behind us would," says Dr. Inglis, "have been an insult to our entertainers." If Benjamin's servants were allowed to do the same, the point of giving him a five-fold "mess" becomes at once apparent.

The above *menu* is by no means an unattractive one; and when we hear that plantains, bread-fruit, yams, sweet potatoes, sugar-cane, and last, but not least, turtle, are common articles of diet, we feel that life on these islands is by no means without its advantages. The scenery of Aneityum, also, is described by Bishop Patteson as extremely beautiful, and the climate is delightful, though, alas! treacherous, fever and ague being common complaints among the New Hebrideans.

"A missionary," says Livingstone, "must be Jack-of-all-trades;" and Dr. Inglis would seem to have laid this maxim to heart, for we find him not only preaching the Gospel, but building houses, making laws and administering justice (mostly through the chiefs), and dispensing medicines. He is also fond of natural history, and when we come to his remarks upon the gigantic shell-fish of Aneityum we can but compare him with that excellent "praste," Father O'Flynn, who could range at will—

"Out of mythology into thayology,
Troth, and conchology, if he'd the call!"

With regard to Dr. Inglis' dispensing duties, our readers will be interested to hear that his great idea has always been to avoid pauperising the natives, and that he therefore arranged a system by which each teacher or chief should collect week by week so many baskets of taro among the people of his district, and send in this contribution to the missionaries, every man being then at liberty to obtain advice and medicine gratis. The system seems to have worked excellently; the power and influence of the native "sacred men" rapidly declined; and the people came to have unbounded confidence in the medical treatment of the missionaries.

Not the least interesting part of the book is that containing the letters of Williamu, a native of Aneityum, who was taken by the missionaries on a visit to England. His accounts of the sights of London, which made him "weak with wonder," and of the kindness of the Beretani (Britons), who "gave him every day as much as he could eat," are charmingly *naif* and graphic; and when the news reaches him of the death of his wife, his simple sorrow is very pathetic. "I cannot," he says, "write well to you on account of my grief, nor can I explain to you the sorrow of my heart. My wife, Mary Ann, and I lived first together. But after she died, when I looked at Dora, she was just like Mary Ann, and on this account I married her. But she, too, is gone from me, and my heart is very sad."

A curious custom on Aneityum is that of calling attention to one's grievances or wants by committing a depredation, or some other reprehensible action. Dr. Inglis relates that he once found a fine bunch of bananas cut down and left lying in his garden, and that on inquiring of the offender why it had been done, he was told, "Because So-and-so had said so-and-so about me which was not true, and so broke my heart; and I did this that you might ask me about it and reprove them." And again, one of his best native helpers became unbearably rude, throwing stones at passers by, and behaving so extraordinarily that Mrs. Inglis conjectured that he was going out of his mind. The explanation was simply this—that the man wished to be made a teacher instead of a cook. When asked why he did not prefer his request to the missionary, he replied, "Oh, this is our way of doing things," and from that day, although his desire was not gratified for some months, he gave entire satisfaction once more. Is it possible that the havoc made among our crockery by certain little slaveys may be traceable to the same cause? We commend the suggestion to our lady readers.

* "Bible Illustrations from the New Hebrides." By the Rev. John Inglis, D.D. (London: Thomas Nelson and Sons.)

